# Advances in testing for sample manipulation in clinical and forensic toxicology - Part A: urine samples

**DOI:** 10.1007/s00216-023-04711-w

**Published:** 2023-05-05

**Authors:** Dirk K. Wissenbach, Andrea E. Steuer

**Affiliations:** 1 Institute of Forensic Medicine, Jena University Hospital, Jena, Germany; 2 Department of Forensic Pharmacology & Toxicology, Zurich Institute of Forensic Medicine, University of Zurich, Winterthurerstrasse 190/52, CH-8057 Zurich, Switzerland

**Keywords:** Urine, Clinical toxicology, Forensic toxicology, Sample manipulation testing

## Abstract

In many countries, adherence testing is used to monitor consumption behavior or to prove abstinence. Urine and hair are most commonly used, although other biological fluids are available. Positive test results are usually associated with serious legal or economic consequences. Therefore, various sample manipulation and adulteration strategies are used to circumvent such a positive result. In these critical review articles on sample adulteration of urine (part A) and hair samples (part B) in the context of clinical and forensic toxicology, recent trends and strategies to improve sample adulteration and manipulation testing published in the past 10 years are described and discussed. Typical manipulation and adulteration strategies include undercutting the limits of detection/cut-off by dilution, substitution, and adulteration. New or alternative strategies for detecting sample manipulation attempts can be generally divided into improved detection of established urine validity markers and direct and indirect techniques or approaches to screening for new adulteration markers. In this part A of the review article, we focused on urine samples, where the focus in recent years has been on new (in)direct substitution markers, particularly for synthetic (fake) urine. Despite various and promising advances in detecting manipulation, it remains a challenge in clinical and forensic toxicology, and simple, reliable, specific, and objective markers/techniques are still lacking, for example, for synthetic urine.

## Introduction

Monitoring of (drug) consumption behavior and regular drug testing to prove abstinence from alcohol, drugs of abuse (DOA), or addictive prescription drugs are implemented in many countries to ascertain drug-freeness, for example, in the context of driving, military, workplace, or doping [1–6]. Various matrices are used for this purpose, with urine and hair being the most commonly used nowadays. Urine is characterized above all by its simple and noninvasive sampling procedure [3, 6–8]. Its moderate detection window for most drugs and/or drug metabolites is well suited for continuous urine testing within short notice. Alternatively, hair has gained massive importance for retrospective consumption monitoring in recent years, mainly because of its noninvasive sampling, non-critical storage at room temperature, and long-term detection window.

Drug screening involves several strategies, with immunoassay prescreens for the most common DOAs being the most straightforward way, particularly when high throughput is desired and prevalence is low. Then, only positive results are typically submitted to further confirmatory analysis by hyphenated techniques such as gas chromatography (GC)–mass spectrometry (MS) or liquid chromatography (LC)-MS [8–10]. With the continuous developments in hardware and more user-friendly software applications, comprehensive or targeted LC-MS or LC-MS/MS screening workflows applying both low and high resolutions (HR) are increasing, sometimes even preceding the use of less specific immunoassay technologies [9, 11–13].

Positive drug tests within an abstinence control treatment program are usually associated with severe legal or economic consequences, leading in the worst case to the loss of a driver’s license or workplace. Thus, drug abusers may be highly motivated to manipulate their samples to obtain negative drug test results. Depending on local regulations, samples are defined as negative when drugs are not detected, but more often, if drug concentrations fall below a specific, pre-defined cut-off value. As such, deliberately decreasing a drug concentration below the cut-off might already be sufficient to obtain a negative drug test.

Manipulation strategies in urine samples are manifold [5, 8, 14], and numerous products are commercially available. Adequate testing not only for drugs but also for tampering attempts is increasingly challenging for toxicological laboratories.

The present critical reviews aim to provide an overview of current manipulation strategies applied to urine (part A) and hair specimens (part B) and their current (routine) detection methods, as well as to highlight and critically discuss recent advances in research and their application. For part A, PubMed was searched for new and innovative technical developments or biomarkers within the past 10 years with the following search terms: “sample adulteration AND forensic,”,“sample adulteration AND clinical,” “biomarker AND adulteration,” “urine AND adulteration,” “urine AND tampering,” “fake urine AND forensic,” “fake urine AND clinical,” “synthetic urine AND forensic,” “synthetic urine AND clinical,” “fetish urine AND forensic,” and “fetish urine AND clinical.”

## Ways of urine adulteration

Even if the prevalence of urine sample manipulation may vary on the region and sample cohort, such sample manipulations are of serious concern and have been evaluated for years [5, 15–21]. Different approaches of urine adulteration/ manipulation to avoid a positive drug test result are described and already extensively discussed. Those attempts may influence either the screening or the confirmation analysis results or both of them [5].

As given in Table 1, one strategy (I) of sample adulteration/manipulation is to undercut the limits of detection/cut-offs of the corresponding (pre)screening or confirmation test. One possibility to reach that goal is to dilute the urine sample either in vivo or in vitro. Concerning an applied cut-off decision, this will lead to true negative results. Another possibility to undercut the limits of detection is the degradation of the corresponding drug itself using different reactive reagents and/or pH, which will also lead to true negative results. A second strategy (II) is to manipulate the analysis of the specimens itself by hampering the analytical approaches. For example, liquid–liquid extractions, but also immunochemical detection, can be influenced by adding detergents to the urine sample. Those detergents may also interfere with the chromatographic system of confirmation analysis. Those approaches will cause false-negative screening results. A third strategy (III) is to substitute the urine sample. For those purposes, different liquids are commonly applied. Most easily, the urine sample is substituted with a liquid such as apple juice. More advantage the sample is substituted with synthetic urine, clean human or animal urine. More detailed information on specific actions to implement strategies I to III is given in the following subchapters.

**Table 1:** Strategies for urine manipulations, methods, probable drug test result, and examples

Strategy/methods		Probable drug test result (if manipulation is undetected)	Examples
Undercutting the limits of detection/cut-offs (screening and/or confirmation)	By dilution	True negative	[22]
	By chemical adulteration	True negative	[21, 23, 24]
Hampering of the analytical approaches (screening and/or confirmation)	By chemical adulteration	*False-negative*	[21, 25, 25–27]
Substitution	By other liquids	True negative	[28, 27]
	By synthetic urine	True negative	[20, 29]

### Dilution

Dilution probably is still one of the most applied urine tampering methods [15, 19]. Those attempts will be uncovered by the determination of urinary creatinine levels and/or specific gravity using current procedures (see below). Depending on national and international guidelines samples with abnormal creatinine levels and/or specific gravity will be classified as “invalid” or “substituted” and rejected for drug testing. Recently, Feldhammer et al. published a case report on a diluted urine sample with unusually low creatinine levels and a specific gravity of 1.004. Suspicious sediment was found in this urine sample. Hydrocodone, but no corresponding metabolites, was detected in that sample. The authors concluded that the urine sample was diluted and additionally manipulated by crushing a hydrocodone pill to mask the dilution [22].

While there is an ongoing debate on certain creatinine levels and specific gravity cut-offs, the question arose if supplementation may mask a heavy in vivo or in vitro dilution. Franz et al. found that the ingestion of creatine may increase urinary creatinine levels, which may allow to mask a dilution. For “light-colored” urine samples showing creatinine levels above the recommended threshold of 20 mg/dL, the authors recommended quantifying urinary creatine levels, determination of the specific gravity, and correlation of the obtained creatinine concentrations and specific gravity to exclude sample manipulation [30].

### Chemical adulteration

Chemical adulteration, e.g., by household chemicals, is known to disturb different immunoassay test systems/test principles as well as chromatographic confirmation analysis [5]. In order to describe these disturbances for a competitive enzyme-linked immunosorbent assay (ELISA) (pre)screening, Olivieri et al. adulterated spiked urine samples with seven different compounds and ascending concentrations. The authors showed that bleach, sodium hydroxide (NaOH), vinegar, and sodium nitrite had the most significant negative impact on the cannabinoid, cocaine, and amphetamine assay. Those results were somehow in accordance with literature data for other immunoassay systems [5]. It was shown that several adulterants—even at low concentrations—may generate false-negative ELISA results [25].

Another study investigated the effects of household chemicals like acids, alkalis, oxidizing compounds, some surfactants, and glutaraldehyde on immunoassay test strips. Confirmed drug-positive urine samples were adulterated up to a concentration of  ~ 40% (v:v) or the corresponding solubility, and strip tests were performed. Different results were obtained depending on the test strip assay and the adulteration agent. While visine eyedrops do not show any change in the detection rate of adulterated urine samples, the NaOH-containing product led to “invalid” test strip results. Overall, acids, especially vinegar, showed to be a potent adulterant for the investigated test strip system, leading most of the time to negative screening results. The cannabinoid test was more susceptible to sample adulteration than the cocaine test [23].

A similar adulterant panel was tested by Matriciani et al. for cloned enzyme donor immunoassays (CEDIA) and Diagnostic Reagent Inc. (DRI®) detection and common DOAs such as oxazepam, amphetamine, 3,4-methylenedioxy-N-methylamphetamine (MDMA), tetrahydrocannabinol carboxylic acid (THC-COOH), ethyl glucuronide (EtG), morphine, 2-ethylidene-1,5-dimethyl-3,3-diphenylpyrrolidine (EDDP), and benzoylecgonine. Oxidizing agents were most effective in producing false-negative results for benzoylecgonine, EDDP, EtG, and morphine. Once again, the screening for THC-COOH was influenced mainly by several adulterants [24].

The effect of different concentrations of sodium hypochlorite (NaOCl) on CEDIA-based screening and chromatographic confirmation analysis of MDMA was investigated by Pham et al. False-negative CEDIA results were found, but only for high reagent concentrations. The authors pointed out that for those samples, strong negative reading results were obtained by the CEDIA assay, which may be used as an indicator for a sample adulteration [26]. Different case reports on sample adulteration using detergents/soaps have been published recently. The mechanisms of the interferences were different for the applied analytical workflows and/or utilized reagents/reagent kits. Feliu et al. found that the immunoassay screening revealed false-positive results by a soapy adulterant and reported this to the immunoassay manufacturer [28].

Recently, CEDIA-based and two different dipstick immunoassays for cannabinoids and amphetamines/MDMA were tested for interferences by household chemicals [21]. The impact on adulteration was different for the investigated immunoassays and indeed differed for the adulterant and its concentration. The authors found opposite findings for the CEDIA test parameters cannabinoids and amphetamine/MDMA. While Olivieri et al. described a minor decrease of the CEDIA cannabinoid signal by acid, Aydogdu and Akgur observed no influence. In contrast, Aydogdu and Akgur found an impact of acetic acids on amphetamines/MDMA. This effect was not seen by Olivieri et al. [25]. Concerning the two different dipstick immunoassays, it was shown that only for NaOH, “invalid” test results were found by both dip cards (cannabinoid test field). Using higher concentrations of bleach, false-negative results were found by both dipsticks for cannabinoids. Acetic acid also showed an impact (false-negative) on the cannabinoid test field of both dipsticks [21].

Gmeiner and Geisendorfer described two cases of urine adulteration with surfactants, which led to massive foam formation and interference with the chromatographic GC-MS procedure. Performing GC-MS analysis after enzymatic hydrolysis, liquid–liquid extraction, and derivatization, they found no hint of adulteration for one of both samples. However, the second sample showed significantly lower efficiencies for hydrolysis and derivatization [27].

An exciting case report on adulteration with an alcoholic beverage was published by Segura et al. summarizing a doping case with steroids in the late 1990s [31].

#### Substitution

Dependent on the sample cohort group and region, manipulation by substitution is probably the second most urine adulteration method [19–21]. Instead of substitution with other fluids like toilet water, fruit juices, and soap, substitution with synthetic urine, aka fake urine or fetish urine, has become more popular over the last years [20, 27, 29, 32–35].

## Current procedures to detect urine manipulation attempts

Even if sample manipulation test strategies/specimen validity testing (SVT) like optical inspection, specific gravity, creatinine level, and pH determination is obliged in different international and national guidelines for urine analysis for certain drug testing scenarios, testing for sample manipulation represents a major challenge for clinical or forensic laboratories [5, 16, 36]. Kirsh et al. recently published a survey among members of the American Society of Addiction Medicine regarding their knowledge, understanding, and practices in urine drug testing, where 79% of 365 (34% of all invited) participants considered manipulation testing important [37]. SVT should be specific and sensitive but also time- and cost-effective. Ideally, workflows would allow simultaneous detection of all manipulation attempts in the very same run with drug detection. With ever-improving analytical technologies, sensitivity is often no longer the limiting factor. However, detecting fake or substituted urine and identifying chemical adulteration remains an issue.

With creatinine, a reasonably well-performing marker for in vivo and in vitro urine dilution exist [32, 38–40], despite discussions on reasonable cut-off limits to consider a sample as diluted [17, 40, 41].

Lin et al. investigated over 21,000 submitted drug test samples from workplace drug testing and court settings using an SVT panel. This panel consisted of determining creatinine, pH, and specific gravity for some samples but not screening for oxidizing compounds and/or substances that are not normal constituents of urine. The authors found a mean 5-year prevalence of urine manipulation of  ~ 1.0% for workplace drug testing and  ~ 3.8% for court setting samples. Even if the proportion of urine dilution, urine substitution, and other urine manipulations differed for both sample cohorts, urine dilution was the most prevalent sample manipulation for both cohort groups [19].

In their study on the effect of chemical adulteration on ELISA detection, Olivieri et al. also evaluated the performance of two commercially available adulterant test strips systems. In addition to pH, both test systems could detect and semi-quantify creatinine, nitrite, and glutaraldehyde. The AC6 adulteration test strip also determines chromate and oxidants, while for the In7 adulteration test strip, reaction pad for specific gravity, bleach, and pyridium chlorochromate (PCC) is present. Both test systems were able to detect oxidizing compounds accurately at different concentrations. Overall, the In7 test strips were more sensitive to detect the presence of an adulterant, primarily due to the specific gravity, which was already described in the early 2000s [42, 43]. However, the study showed that both test systems could not detect all adulterants at such low concentrations, which may influence ELISA screening assays negatively [25].

Aydogdu and Akgur used In7 test strips to screen for adulteration by household chemicals, such as NaOCl, NaOH, sodium carbonate, and acetic acid, but also for benzalkonium chloride, an ingredient of pharmaceutical products. The strip system detected adulteration by “abnormal” pH results for acetic acid. Higher concentrations of bleach were detected by the bleach reaction pad, while lower concentrations were uncovered. “Abnormal” results for glutaraldehyde and PCC were also found for higher concentrations of bleach and NaOH. In summary, only for 5 out of 13 adulterated samples the adulteration test systems provided hints of an adulteration [21].

The possibility of detecting sample adulteration with household chemicals by a general biochemical urine test strip system was also investigated by Rajsic et al. This study used a test strip system covering pH, specific gravity, nitrite, and ketones, but also more specific compounds such as ascorbic acids, glucose, protein, bilirubin, urobilinogen, blood, and leukocyte cells. Five out of nine adulterations were detected by the system, mainly by pH value or by an “invalid” result from another test field, such as “protein.” The authors concluded that SVT should not only be performed by biochemical tests but also by visible inspection and warmth of the collected sample [23].

Matriciani et al. evaluated a CEDIA-based sample check for detecting chemical adulterants such as acids, alkalis, oxidizing agents, and detergents. Acids and alkalis were efficiently detected by the CEDIA sample check. However, even if the CEDIA test successfully detected a higher concentration of an adulterant, it failed to a certain amount for those samples with low adulterant concentrations [24]. Aydogdu and Akgur found a similar or even lower performance of the CEDIA-based sample check. In their study, only 20% of NaOH was detected by this adulteration test, while lower concentrations of NaOH, bleach, and acetic acids were not recognized by this assay [21].

## New or alternative strategies for the detection of urine sample manipulation

Progress has been made in recent years to evaluate alternative approaches to screen for urine manipulation attempts. An overview of the chosen techniques, biomarkers, or strategies, including details on the analytical parameters, is provided in Table 2. In general, these can be divided into three different categories, technical advances for improved detection of established urine validity parameters (1), as well as direct (2) and indirect (3) approaches to screen for new detection markers of urine adulteration or substitution. Direct approaches have focused on biomarkers that would directly and unambiguously identify a urine sample as having been manipulated, e.g., through markers that are only present in fake urine. Indirect approaches, on the other hand, aimed at finding (new) urine validity parameters that would classify a sample as suspicious. A major focus in recent years has been on small (endogenous) molecules (molecular weight  < 1000 Da), given their ease in analysis, similar to drug screening and quantification methods in clinical and forensic toxicology. The experiments performed to identify biomarkers can be described broadly as metabolomics. Applications of targeted and untargeted metabolomics in clinical and forensic toxicology have recently been published [44–48], including sample manipulation. Often, more sophisticated MS approaches were chosen for that purpose. While some of these techniques might be too complex for routine high-throughput application, particularly in laboratories relying on initial immunoassay prescreens, they still provide the basis for future developments of point-of-care or immunoassay tests on new biomarkers.

**Table 2:** Method details of new approaches to screen for different urine manipulation attempts

Manipulation	Matrix	Biomarker/technique	Sample preparation	Analytical device	Settings	Data evaluation	
Dilution	Urine	Creatinine	None	3D-μPAD Three-dimensional microfluidic paper-based device	Three colorimetric reagents: - 3,5-dinitrobenzoic acid - Picric acid - Nessler’s reagent	Color change - Visually - iPhone camera - RGB values	[49]
		Creatinine (method comparison)	PP with ACN (1:3, v/v)	LC-MS/MS Sciex 5500 QTrap	SeQuant ZIC®-HILIC - 5 mM NH4Ac buffer (pH 5.72), 0.1% FA - ACN, 0.1% FA ESI- MRM	Quantification Five-point calibration	[39]
			None	Spectrophotometry Beckman Coulter AU 480	Jaffe reaction	One-point calibrator	
			None	Point-of-care test Protzek	Indicator dye by a creatinine/copper complex	Automatic readout system	
		Specific gravity	None	Digital refractometer PalmAbbe™ PA202X		EP Evaluator StatisPro™ 2.51 Microsoft Excel 2010	[50]
Dilution/adulteration	Urine	Creatinine, uric acid	Dilution with H_2_O, filtration	LC-MS/MS API 3200 QTrap	ODS column Scherzo SM-C18 - 0.2% formic acid - ACN ESI + /ESI- MRM	Quantification Seven-point calibration	[51]
Adulteration Surfactant/liquid soaps	Urine	Characteristic peak patterns: long-chained aliphatic hydrocarbons (CH_2_-increments)	evaporation to dryness MSTFA/TMSI derivatization	GC-MS ITQ 1100 MS	RTX-5 EI (70 eV) Full-scan	XCALIBUR software	[27]
Adulteration NaOCl	Urine	MDMA oxidation products (Chloro-MDMA)		LC-MS/MS Agilent 6460 QQQ	XBridge C18 column - 2 mM NH4COOH in H2O - ACN ESI + Full-scan, MRM	Mass spectral comparison/interpretation	[26]
Adulteration KNO_2_	Urine	Oxidation products of 6-MAM Morphine Morphine 3-glucuronide Morphine 6-glucuronide Codeine	Centrifugation Filtration	LC-MS Agilent 6460 QQQ	Agilent Zorbax Eclipse Plus C18 - 20 mM NH4COOH (pH ≈ 6.3) - ACN ESI + Full-scan, PIS, MRM	Mass spectral comparison/interpretation NMR interpretation	[52, 53]
Adulteration PCC	Urine	Oxidation products of 6-MAM Morphine Morphine 3-glucuronide Morphine 6-glucuronide Codeine Codeine 6-glucuronide	Native Derivatization BSTFA/TMCS	LC-HRMS Agilent 6510 QTOF GC–MS Agilent 5975C Inert XL NMR Bruker Avance III 600 MHz NMR	10% ACN in 20 mM NH4COOH Agilent HP-5MS capillary column EI-MS Full-scan Solvent CDCl_3_ 1D ^1^HNMR 2D ^1^H-^1^H COSY, ^1^H-^13^C HSQC, ^1^H-^13^C HMBC		[54, 55]
Adulteration KNO_2_	Urine	Untargeted search	Dilution with ACN (1:3, v/v)	HPLC-HRMS Sciex 6600 qTOF	XSelect HSST RP-C18 - 10 mM NH4COOH in H2O, 0.1% FA - Methanol, 0.1% FA ESI + Full-scan + DDA	XCMSplus Metabo Analyst 3.0 Peak View/chemspider Identification (NIST, Metlin, HMDB)	[56]
Adulteration KNO_2_ PCC H_2_O_2_ I_2_ NaOCl	Urine	Uric acid (UA), HIU, HIC Methyl UA 1,3- /1,7-dimethyl UA Indolylacroylglycine (IAG) Histidine Methylhistidine Imidazole lactate Methylimidazole lactate acetylneuraminic acid Dimethyllysine Trimethyllysine	PP with ACN (1:3, v/v)	HPLC-HRMS Sciex qTOF 6600	XSelect HSST RP-C18 - 10 mM NH4COOH in H2O, 0.1% FA - Methanol, 0.1% FA ESI + Full-scan + DDA	Multiquant Quantification Six-point calibration (in synthetic urine)	[57, 58]
		Untargeted approach/machine learning model	PP with ACN (1:3, v/v)	HPLC-HRMS Sciex qTOF 6600	XSelect HSST RP-C18 - 10 mM NH4COOH in H2O, 0.1% FA - Methanol, 0.1% FA ESI + Full-scan + DDA	Progenesis QI RStudio (version 1.2.1335 Keras within R ANN LIME	[59]
Substitution synthetic urine	Urine	Unknown	None	Synthetic UrineCheck™ dipstick test (Sciteck)		Visual, color	[34]
		Unknown	None	Synthetic UrineCheck™ dipstick test (Sciteck)		Visual, color	[35]
		Unknown	None	Beckman‐Coulter AU 2700 autoanalyzer SYN 750 (Sciteck)		Absorbance Negative – synthetic urine Positive—normal urine	[33]
		Ammonium magnesium phosphate Calcium phosphate Ammonium urates and unknown others	None	Axiom Test True synthetic urine assay		Absorbance Combination of specitifc gravity, pH, and test result	[60]
		Untargeted Search	Not given	UPLC-HRMS Xevo® G2 TOF	ACQUITY® HSS C18 - 5 mM NH4COOH - ACN	not given	[29]
		Benzisothiazolinone triethylene glycol (E3G) tetraethylene glycol (E4G)	Dilution with MeOH (1:3, v/v)	LC-MS/MS Sciex 5500 QTrap	ACQUITY® HSS T3 - 10 mM NH4Ac, 0.1% FA - ACN ESI + MRM	Quantification Calibration in synthetic urine	
		Tetrapropylene glycol Pentapropylene glycol Hexapropylene glycol Heptapropylene glycol Octapropylene glycol Nonapropylene glycol Decapropylene glycol Undecapropylene glycol Within untargeted screening	PP with ACN (1:5, v/v)	LC-MS/MS LTQ XL linear ion trap	EC100/3 Nucleoshell RP18plus - 10 mM NH4COOH, 0.1% FA - ACN, 0.1% FA ESI + , DDA	TF ToxID 2.1.1 Library search Qualitative	[20]
Substitution Human urine/dilution	Urine	Triplex STR analysis	DNA extraction - Quick-DNATM kit - Urine-DNA IsolationTM kit	Electrophoresis with fluorescence detection			[61]
Substitution	Urine	Creatinine Uric acid Methylhistidine Normetanephrine Urobilin Cotinine Theophylline, theobromine	Dilution with MeOH (1:3, v/v)	LC-MS/MS Sciex 5500 QTrap	ACQUITY® HSS T3 - 10 mM NH4Ac, 0.1% FA - ACN ESI + MRM	Quantification Calibration in synthetic urine	[29]
		Phenylalanine Tryptophan Propionyl‐carnitine Butyryl‐carnitine Isovaleryl‐carnitine Hexanoyl‐carnitine Phenylacetylglutamine Heptanoyl‐carnitine Octanoyl‐carnitine Indoleacetylglutamine Within untargeted screening	PP with ACN (1:5, v/v)	LC-MS/MS LTQ XL linear ion trap	EC100/3 Nucleoshell RP18plus - 10 mM NH4COOH, 0.1% FA - ACN, 0.1% FA ESI + , DDA	TF ToxID 2.1.1 Library search Qualitative	[20]
		35 endogenous compounds including: Phenylalanine Tryptophan Propionyl‐carnitine Butyryl‐carnitine Isovaleryl‐carnitine Hexanoyl‐carnitine Phenylacetylglutamine Heptanoyl‐carnitine Octanoyl‐carnitine Indoleacetylglutamine	PP with ACN (1:4, v/v)	LC-HRMS Exactive Focus Hybrid- Quadrupol-Orbitrap	Nucleoshell RP 18 plus ESI + Full-scan, HRMS^2^ fragment spectra	Library search (mzcloud, massbank, HMD, Metlin, NIST, MWW, (conjugated) AC library)	[62]
Substitution Animal urine	Urine	Untargeted search	cEntrifugation, mixing with D_2_O	NMR 600-MHz spectrometer Agilent	600.167 MHz (14.1 T) Pre-saturation pulse sequence 1.52 s relaxation delay 1.998 s acquisition time 8 min total acquisition time	SIMCA-P + 12.0 software Chenomx NMR suite7.1	[63]
Substitution Human urine	Urine	Low-molecular-mass polyethylene glycols (orally ingested prior to urine delivery)	Centrifugation	LC-UV UVD 170S UV detector	Nucleosil 100 C pre-column Nucleosil 100 C analytical column 44% MeOH; 56% H_2_O (isogratic)	Quantification Four-point calibration	[64]

Abbreviations: *LIME*, local interpretable model-agnostic explanations

### Dilution

### Technical innovations

Creatinine is almost certainly the most popular and most often routinely measured urine validity parameter to screen for manipulation attempts. Musile et al. developed an origami paper-based microfluidics technology (3D-μPAD) as an on-site device for creatinine determination. The device included three colorimetric reactions based on picric acid, 3,5-dinitrobenzoic acid, and for the first time, Nessler’s reagent, with color detection by a built-in smartphone camera. The device was evaluated with 48 urine samples. Nessler’s reagent was superior to picric acid-based and dinitrobenzoic acid-based reagents and classified 18 urine samples as diluted in line with the reference enzymatic assay [49]. Lugingbuehl and Weinmann tested a point-of-care testing device to determine urinary creatinine concentration by a copper ion complexation and dye indication. They compared the obtained creatinine concentrations to those determined by multiple reaction monitoring (MRM)-based HILIC LC-MS/MS and by colorimetric (Jaffe reaction) spectrophotometry. The two laboratory methods were more reliable than the point-of-care device. However, the authors appreciated the point-of-care testing device for providing an automated interpretation of the on-site test results. They concluded that this new device might be helpful for on-site creatinine testing [39].

Determination of specific gravity to assess hydration status and specimen validity is either performed by test strips or, more typically, using manual or digital refractometers—the latter measure a solution’s refractive index, which is affected by solute concentration. From a clinical laboratory perspective, only a few comprehensive performance evaluations are available. Therefore, Wyness et al. recently performed an analytical validation of a handheld digital refractometer. A strong correlation in accuracy compared to manual refractometry could be shown, and, in addition, a linear correlation could be observed between specific gravity and osmolality. Overall, the handheld refractometer proved to be a simple, accurate, and fast tool to measure specific gravity [50].

### Chemical adulteration

Progress to test for sample adulteration mainly focused on indirect methods. Only one case report, published by Gmeiner and Geisendorfer, used a direct approach to detect urine adulteration with liquid soap in two cases. Following initial suspicion, given an unusually high amount of foam above the liquid phase of the urine samples, they confirmed a urine manipulation with surfactants by detecting corresponding adulterant-specific signals by GC-MS after TMS derivatization [27].

### Indirect approaches: oxidation markers of drugs

**Fig. 1:**
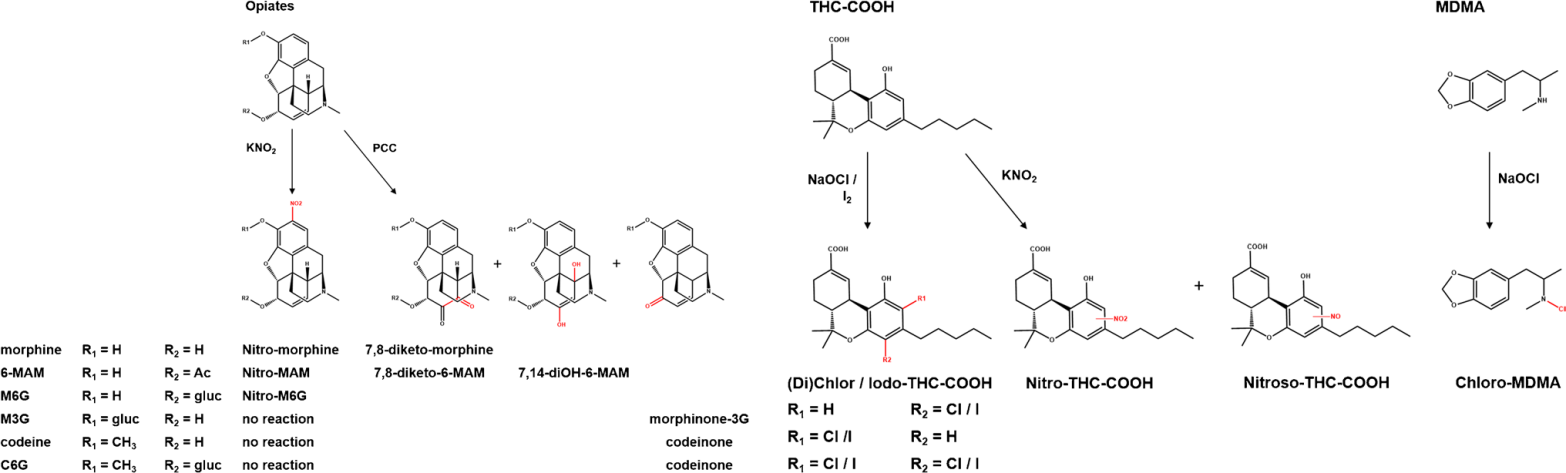
Drug oxidation products of opiates, THC-COOH, and MDMA with different adulterants (KNO_2_, PCC, NaOCl, and I_2_) as described in references [5, 26, 52–55, 76]. 6-MAM, 6-monoacetylmorphine; M6G, morphine 6-glucuronide; M3G, morphine 3-glucuronide; C6G, codeine 6-glucuronide; morphinone 3G, morphinone 3-glucuronide

While routinely used methods are more or less capable of identifying a sample as chemically adulterated, they do not reveal the types of drugs consumed. Therefore, one strategy in improved adulteration testing involved screening for and identification of drug oxidation products. As the drug itself is potentially no longer detectable due to oxidative degradation, detecting drugs in their oxidized forms would demonstrate the act of adulteration and the specific drug taken. This approach was extensively discussed in Fu’s latest review on sample adulteration in 2016 [5]. To the best of our knowledge, no new research has been published in the last 6 years describing drug oxidation products as potential combined markers of drug intake and urine manipulation. The present review will, therefore, only briefly summarize the former findings. The following drugs and adulterants have been studied: MDMA, THC-COOH, morphine, morphine-3-glucuronide, morphine-6-glucuronide, 6-monoacetylmorphine, codeine, and codeine-6-gluruconide with NaOCl, iodine (I_2_), potassium nitrite (KNO_2_), and PCC. An overview of the respective combinations and resulting oxidation products identified is given in Fig. 1. The stability of the oxidized drugs represents a critical issue for their general applicability as adulteration markers and needs to be considered before screening for these markers. Overall, this approach is considered ideal for a confirmation test following easier strategies for adulteration detection as it combines the proof of drug intake and adulteration. Still, identification of such stable oxidation products is required for each drug adulterant combination, which is work- and time-intensive. As a screening method, it will fail in case of newer designer drugs or prescription drugs where knowledge of oxidation products does not exist (yet).

### Oxidation of endogenous compound markers

**Fig. 2:**
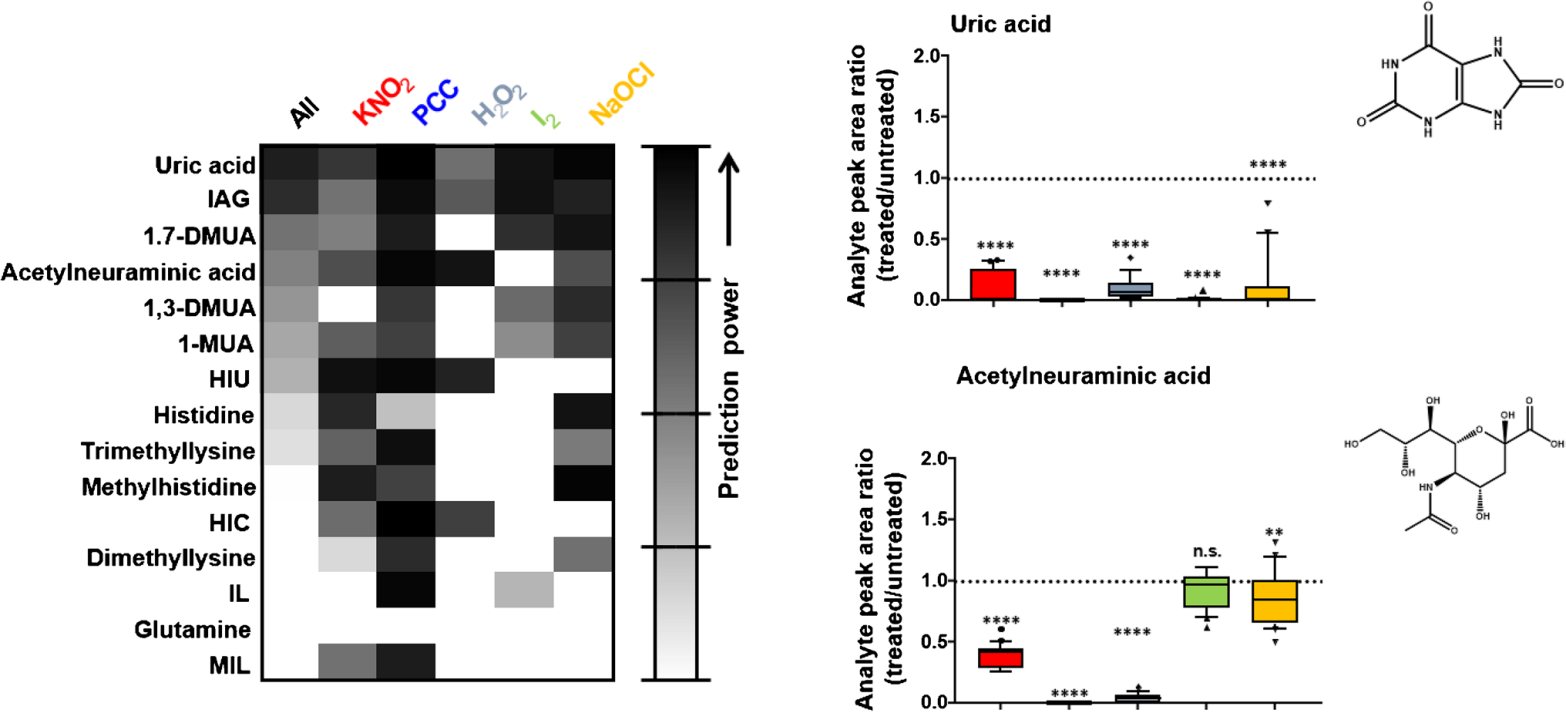
Heat map (left) of the area under the curve (AUC) for potential endogenous biomarkers calculated from receiver operating characteristics (ROC) curve analysis for all adulterants vs. control (all, *n* = 100) or every single adulterant vs. control (*n* = 20 each). The closer to one (black) the AUC is, the better the predictability of an authentic sample as being adulterated or not. Box plots (right) of analyte peak area ratios of paired treated over untreated samples (*n* = 20, *y*‐axis) for the different adulterants (*x*‐axis). The dotted line represents a ratio of 1, indicating no influence of the adulterant. Data below 1 indicate degradation, those above 1 formation through the adulteration reaction. Statistical tests were performed using one-sample *t* tests (theoretical mean 1, n.s. *p* > 0.05; ** *p* < 0.01; *** *p* < 0.001; **** *p* < 0.0001). Figure(s) adapted from Steuer et al. [58] with permission from Wiley

Likely, adulterants that can oxidize drugs will also react with endogenous compounds in human urine. Identifying compounds subject to adulteration in terms of degradation or neo-formation might allow their use as potentially indirect adulteration markers independent of previous drug consumption. First investigations used an untargeted metabolome approach to screen for such changes in small endogenous molecules induced by chemical adulteration with the oxidant KNO_2_. Several promising markers were identified, e.g., degraded products like uric acid and some of its (di)methylated derivatives, (methyl)histidine, or acetylneuraminic acid, as well as the neo-formation of 5-hydroxyisourate, the oxidation product of uric acid. While identifying compounds degraded through oxidative treatment was relatively straightforward, annotating newly formed oxidation products remained challenging as their mass spectra were lacking in common databases for endogenous compounds [56]. Biomarkers formed only through adulteration represent the most accessible and reliable markers. Unfortunately, the only marker that fulfilled that criterion, namely, 5-hydroxyisourate, turned out unstable at room temperature after 1 day. Following validated quantification, degradation of uric acid (proposed cut-off  < 84 μmol/mmol creatinine) and indolylacroylglycine (IAG) (proposed cut-off  < 0.45 μmol/mmol creatinine) indicated promising performance (specificity and sensitivity  > 0.9) for KNO_2_ [57, 58] as well as for the other adulterants (PCC, hydrogen peroxide (H_2_O_2_), NaOCl, I_2_) [58] summarized in Fig. 2, though. Acetylneuramic acid showed weaker prediction power for any oxidative treatment but good classification properties, specifically for PCC and H_2_O_2_.

Still, the full potential of the untargeted metabolome approach could not yet be exploited, given the bottleneck of reliable identification of newly formed biomarkers. To overcome this, Streun et al. evaluated machine learning, specifically an artificial neural network (ANN), to classify chemically adulterated urine samples based on their LC-HRMS acquisition data [59]. An equally divided training set of 500 treated (KNO_2_, PCC, H_2_O_2_, I_2_, NaOCl, different conditions) and untreated urine samples were used to build the final ANN. In addition, a local interpretable model-agnostic explanation (LIME) approach was used to highlight MS peaks (features) with the best discrimination properties but without further peak annotation. The model was validated with an independent test set of 200 urine samples, and mean accuracy was determined to be about 95%. Compared to the single biomarker uric acid and indolylacryloylglycine, the ANN revealed superior performance [45, 59]. Without further identification of the discriminating MS features, transferring the model to other laboratories will be impossible. Indeed, more research is needed in real applications of these markers across different laboratories before the final evaluation of their prediction properties.

## Substitution

### Direct approaches

About 5 years ago, a commercial colorimetric assay was introduced that should be able to reveal a specimen as synthetic urine. The test is available as dipsticks or liquids for autoanalyzers and uses a specific indicator for detecting a factor indicative of fake urine [65]. However, this target “analyte” or “factor” is not disclosed by the manufacturer, and as such, the underlying test principle remains unknown. Kim et al. were the first to evaluate the on-site test strips with 116 random urine samples and nine synthetic urine products. They found that the fake urine samples were detected with high sensitivity (9/9) and that diluted specimens could be falsely detected as synthetic urine [34].

In contrast, Vikingsson et al. found color changes in the dipstick tests (synthetic urines lighter in color than authentic ones) challenging to interpret and to discriminate fake from authentic urine samples [35] unambiguously. According to the manufacturers, autoanalyzer testing is superior to dipsticks [65]. An extended study by Silva et al. with the same test principle but using an autoanalyzer device came to similar conclusions as those published by Kim et al. Again, all synthetic urine products were correctly identified (5/5). Similar percentages as in the former study of random urine samples (5.4%, total of 843 urine specimens) were determined as potentially substituted (fake) urine. Most indicated low creatinine values, though, which would allow the assumption that in some cases, the unknown target analyte of the assay could be diluted to such an extent that it would (falsely) indicate synthetic urine [33]. However, without knowledge of the test principle or target analyte, reliability assessment of the test, also with potentially changing synthetic urine products entering the market, remains problematic. In their work, Mina et al. used a commercial colorimetric test system for the detection of synthetic urine [60]. Based on the vendors’ information, multiple urine constituents/compound classes such as calcium phosphate and ammonium urates but also others are used in combination with specific gravity and urine pH. Five different synthetic urine samples and a cohort of 2000 samples were tested by this assay. Approximately 4,4% of the samples were marked as potentially synthetic urine. However, the authors stated that those colorimetric results should be confirmed by a chromatographic system.

In a metabolome-like experiment, Goggin et al. used eight synthetic urine samples from local (Minneapolis) and national shops and compared LC-TOF-MS datafiles to data from authentic urine samples. Identification of peaks detectable in fake but not authentic urine samples resulted in two possible synthetic urine markers, benzisothiazolinone, and ethylene glycols, triethylene glycol, and tetraethylene glycol. However, these markers were detected in only two of the eight included synthetic urine products, while the remaining six did not indicate any unusual components. Application of these markers to  > 3800 routine urine samples led to the identification of only eight presumably substituted, synthetic urine samples, as four tested positive for BIT and four others for ethylene glycols, respectively. These markers occurred in none of the urine samples considered authentic and not manipulated [29]. However, a similar study by Kluge et al. on new urine validity markers in general detected the proposed ethylene glycols several times in authentic urine samples that otherwise showed no indication of manipulation. They considered these ethylene glycols as contamination of specific urine-sampling devices rather than a marker for synthetic urine. Instead, three out of 550 tested urine samples showed patterns of polypropylene glycols, similar to the ones observed in fake urine samples purchased locally (Germany). However, neither endogenous biomolecules nor ethylene glycols were present in these commercially fake urine products [20]. It is not surprising that various synthetic urine products or batches differ in composition, depending on location and time of purchase. Up to now, no specific, universal biomarker for synthetic urine products exists. Further studies are necessary, including higher numbers of synthetic urine products from different (international) origins produced at various periods to screen for such markers.

Detecting replacement attempts with another person’s urine represents a more significant challenge than substituting synthetic, at least for typical toxicology laboratories. DNA analysis focusing on short tandem repeats (STR) is the method of choice in forensic genetic analysis to identify individuals unequivocally and has also been applied in sports doping manipulation testing, as reviewed in detail elsewhere [66, 67]. Pires et al. have successfully evaluated the simplification of this strategy to only three STRs to identify urine samples either mixed with the urine of a different person or with non-human fluids in volume ratios exceeding 25%. Future perspectives on using this approach, even on-site, depend on the possibility of implementing this method into an integrated microchip system [61]. Recent developments in micro-sampling techniques using, e.g., dried matrix spots, led to increased application of this sample form in drug testing [68–70]. Grignani et al. evaluated the possibility of using dried urine samples as a source of DNA for personal identification purposes. The chosen approach proved valid for individual genetic identification from dried urine samples stored for up to 12 weeks, which could be helpful in anti-doping or drug screening if tampering is suspected [71].

### Indirect approaches

Several strategies have been developed and tested that can be broadly classified as indirect to detect the substitution of urine with synthetic, animal, or other donor human urines: search for new validity parameters, characteristic human and/or animal metabolites, or DNA analysis, including technical innovations.

Two similar approaches focusing on small molecules have been performed by Goggin et al. and Kluge et al., aiming to identify stable endogenous markers as new urine validity parameters. Both approaches, as detailed below, were successfully applied to large sample cohorts and can be considered superior to the routinely used validity parameters. Goggin et al. used a targeted LC-MS/MS method for quantitative analysis of four selected markers in addition to creatinine, namely, uric acid, methylhistidine, normetanephrine, and urobilin, as well as frequently observed constituents of tobacco, coffee, or chocolate, namely, cotinine, theophylline, and theobromine. Those four main biomarkers have been chosen from the urine metabolome project [72] and were selected as they were present in practically all samples in high concentrations and amenable to positive ESI measurements [29]. Uric acid has already been used earlier as a manipulation marker to screen for manipulated urine samples. It could be detected and quantified (in combination with creatinine) in a relatively simple LC-MS/MS method [51]. However, uric acid was already present in four of eight [29] or eight of ten tested commercial products [35], raising questions about its sensitivity as a fake urine marker. Instead, Kluge et al. applied an untargeted, universal LC-MS/MS screening approach to qualitatively detect ten proposed endogenous urinary molecules from different (independent) metabolic pathways: phenylalanine, tryptophane, propionyl-carnitine, butyryl‐carnitine, isovaleryl‐carnitine, hexanoyl‐carnitine, heptanoyl‐carnitine, octanoyl‐carnitine, indole-acetyl glutamine, and phenylacetylglutamine. Each marker except for propionyl-carnitine (67%) could be detected in  > 90% of authentic urine samples, while the average number of biomarkers detected in 544 authentic urine samples was 9.4 (ranging from 3 to 10). Following statistical evaluation (mean number of biomarkers detected minus two times standard deviation), urine samples with less than six marker detections were defined as suspicious. Thus, a wrong classification from possible false-negative results in single compounds, which can statistically occur from certain metabolic diseases or the used data-dependent (DDA) methodology, was minimized. Still, Franke et al. extended the initial screening method into a targeted LC-HRMS approach, including the ten biomarkers mentioned above and 25 more endogenous compounds. With this, the method was extended to use different endogenous biomarkers to detect fake urine samples and other forensically relevant issues, such as biofluid identification [62]. Overall, the described procedures proved highly beneficial in urine validity testing but might be limited by their complexity depending on case numbers, laboratory strategies, and infrastructure. Additionally, the description of new markers bears the risk that manufacturers will adapt their products, as exemplified in the frequently observed detection of uric acid in commercial products. Future research should, therefore, mainly focus on markers or marker mixtures that might be difficult to add to synthetic urine samples, e.g., through high costs or high complexity.

The aforementioned LC-MS(/MS) approaches could also be a versatile tool to detect substitution with animal urine. For instance, phenylacetylglutamine is a human‐specific compound, detectable in all human urine samples [20] but not excreted by different animal species such as dogs, cats, or rats [73, 74]. As an explorative study, quantitative ^1^H- nuclear magnetic resonance (NMR) analysis was applied to metabolome profiling to discriminate between human urine and urine samples of different animal species. Multivariate statistics could distinguish urine samples according to species, and several potential characteristic biomarkers were proposed. However, the study must be considered preliminary, and more confirmatory experiments are mandatory, including different diet habits, time series, and routine application [63].

Jones et al. evaluated the need and utility of a polyethylene glycol (PEG) marker system [64] to unmask urine substitution for an *n* = 55-person cohort. In this study, unique PEG markers were administered orally prior to unobserved urine sample collection for a randomized control and verum group. For the verum group, no side effects were observed. For  ~ 83.5% of the verum samples, unique PEG patterns were found by the LC-MS/MS system. PEG patterns were found for four samples, even if no PEG was administered. For those, the authors discussed labeling and laboratory mixed-ups. No PEG pattern was detected for nine samples of the verum group, even if PEG tracers were ingested. The authors stated that for those inconsistent samples, the PEG system proved applicability by detecting fraudulence and concluded that the PEG marker system might be a valuable tool for detecting sample substitution [75]. However, using a PEG marker system and thus unsupervised urine collection, the potential for (chemical) urine adulteration still exists.

## Summary and critical evaluation

Sample manipulation is regularly observed in clinical and forensic toxicology samples to avoid a positive screening result. Although the prevalence of such adulterations may depend on the sample type, sample cohort, and region, these manipulations should always be kept in mind. Different strategies are described, such as undercutting the limits of detection/cut-off by various mechanisms (mainly dilution and adulteration) or substitution, which may affect (pre-)screening or confirmation analysis. According to international and national guidelines, SVT is performed by different methods for various parameters and cut-offs. While SVT adequately detects dilution, chemical adulteration and substitution are still challenging for commercial SVT test systems. Progress has been made in improving SVT methods by developing/evaluating alternative techniques and direct or indirect approaches, such as detecting oxidized target analytes, endogenous biomolecules, and corresponding patterns. Another research focus was on adulterant-specific compounds. Overall, the techniques and procedures described represent a significant advance in validity testing but may be limited by their complexity and feasibility in routine toxicological laboratories. Based on the given information and with respect to international and national guidelines, the impact on urine adulteration by dilution, chemical adulteration, or substitution may be minimized by supervised urine collection followed by temperature control and visual inspection, analysis of the sample validity by SVTs such as creatinine and oxidative substances, and finally screening for the absence of endogenous biomolecules. In the authors’ opinion, screening for several endogenous biomolecules/bio molecules classes (ideally by chromatographic systems) will lead to more robust results for the detection of synthetic urine instead of the usage of single endogenous biomarkers, which may be easily added to synthetic urine specimens.
